# Effects of weather and air pollution on outpatient visits for insect-and-mite-caused dermatitis: an empirical and predictive analysis

**DOI:** 10.1186/s12889-024-18067-y

**Published:** 2024-02-28

**Authors:** Minghua Xiong, Xiaoping Li, Chao Zhang, Shuqun Shen

**Affiliations:** 1 Business School, Foshan University, Foshan, 528000 China; 2 Business School, Sichuan University, Chengdu, 610065 China; 3 School of Business, Southern University of Science and Technology, Shenzhen, 518055 China; 4 Dermatology Hospital, Southern Medical University, Guangzhou, 510515 China; 5 Research Centre for Innovation & Economic Transformation, Research Institute of Social Sciences in Guangdong Province, Guangzhou, 510000 China

**Keywords:** Weather, Air pollution, Dermatitis, Permutation importance, Outpatient visits

## Abstract

**Background:**

Dermatitis caused by insects and mites, diagnosed as papular urticaria or scabies, is a common skin disease. However, there is still a lack of studies about the effects of weather and air pollution on outpatient visits for this disease. This study aims to explore the impacts of meteorological and environmental factors on daily visits of dermatitis outpatients.

**Methods:**

Analyses are conducted on a total of 43,101 outpatient visiting records during the years 2015–2020 from the largest dermatology specialist hospital in Guangzhou, China. Hierarchical cluster models based on Pearson correlation between risk factors are utilized to select regression variables. Linear regression models are fitted to identify the statistically significant associations between the risk factors and daily visits, taking into account the short-term effects of temperatures. Permutation importance is adopted to evaluate the predictive ability of these factors.

**Results:**

Short-term temperatures have positive associations with daily visits and exhibit strong predictive abilities. In terms of total outpatients, the one-day lagged temperature not only has a significant impact on daily visits, but also has the highest median value of permutation importance. This conclusion is robust across most subgroups except for subgroups of summer and scabies, wherein the three-day lagged temperature has a negative effect. By contrast, air pollution has insignificant associations with daily visits and exhibits weak predictive abilities. Moreover, weekdays, holidays and trends have significant impacts on daily visits, but with weak predictive abilities.

**Conclusions:**

Our study suggests that short-term temperatures have positive associations with daily visits and exhibit strong predictive abilities. Nevertheless, air pollution has insignificant associations with daily visits and exhibits weak predictive abilities. The results of this study provide a reference for local authorities to formulate intervention measures and establish an environment-based disease early warning system.

**Supplementary Information:**

The online version contains supplementary material available at 10.1186/s12889-024-18067-y.

## Introduction

Dermatitis is a multifactorial skin condition with diverse etiologies and clinical presentations. There is a substantial body of evidence indicating that dermatitis caused by insects and mites, such as papular urticaria or scabies, is a remarkably frequent dermatologic issue globally [1–3]. Clinically, papular urticaria manifests as a hypersensitivity response to bites from insects such as mosquitoes, fleas, or bedbugs, resulting in persistent and pruritic papules [4]. Similarly, scabies is recognized as a highly contagious pruritic skin condition caused by infestation with an itch mite [5].

Papular urticaria and scabies are prevalent but usually neglected diseases. According to a survey conducted in 12 cities across China, the incidence of papular urticaria is 5.3% among children aged 0–7 years [6]. Likewise, in Colombia, the prevalence of papular urticaria among children aged 1–6 years is reported to be 20.3% [7]. In France, a study estimates the annual incidence of scabies at 0.3% for all inhabitants and reports an increase in the incidence rate during 1999–2010 [8]. A study in northwest Ethiopia reveals that the prevalence of scabies is 9.3% among schoolchildren [9]. The World Health Organization (WHO) added scabies to the list of neglected tropical diseases in 2017 [10].

Recently, climate changes are speculated for causing dermatitis from insects and mites because warm and moist conditions are more likely to breed Arthropods [11]. As evidenced by a wide range of diseases, temperatures are essential in affecting healthcare demands. Extreme temperatures can increase hospital admissions for psychiatric disorders [12, 13], renal disease [14], cardiovascular disease [15, 16], and malaria [17]. Extreme temperatures can also lead to adverse health outcomes such as mortality, length of stay, and costs [18–20]. Epidemiological evidence has shown that extreme temperatures exacerbate core symptoms of psychiatric and cardiovascular conditions [21]. However, other meteorological factors, such as humidity, precipitation, wind speed, and sunshine duration, vary among patient groups [22]. For example, humidity’s effect on Emergency Department admissions for psychiatric disorders is higher for males [23]. Although many diseases are well-researched in the literature, risk factors for papular urticaria and scabies are less often considered from the perspective of weather conditions.

In addition, air pollution is associated with an increased medical care demand for a variety of diseases. Particulate matter (PM), ozone (${{\text{O}}}_{3}$), nitrogen dioxide (${{\text{NO}}}_{2}$), and sulfur dioxide (${{\text{SO}}}_{2}$) have been extensively studied in the literature. High concentrations of PM can increase hospital admissions for cardiovascular diseases [24, 25], pulmonary diseases [26, 27], and Emergency Department visits [28, 29]. High concentrations of air pollutants are also linked to an increased risk of suicide attempts [30] and suicide deaths [31]. The pollution effects on Emergency Department admissions vary among patient groups, being higher in children [32] and women [33]. ${{\text{PM}}}_{2.5}$ has immediate effects on cardiovascular diseases and delayed effects on respiratory diseases [34]. Unfortunately, risk factors for papular urticaria and scabies are also less often considered from the perspective of air pollution.

Although meteorological and environmental factors are significantly associated with medical care demand, such as hospital admissions, in numerous cases, their effects of stratification, overfitting, and model misspecification might hinder their predictive abilities [35, 36]. Therefore, each factor requires evaluation to confirm their practical applications in healthcare operations, such as predicting patient visits. Chen et al. [37] find that utilizing the information of temperature and humidity, and internet search index in the generalized additive model can improve prediction accuracy for patient visits diagnosed with hand-foot-and-mouth disease. Li et al. [38] use Deep Learning to predict Emergency Department admissions with environmental variables, such as temperatures and air quality index. Wang et al. [39] consider outpatient visits prediction and physician scheduling for mental disorders. They find that including meteorological variables, calendar variables, and time-series variables in the generalized smooth model can improve physician schedules timely due to better prediction of short-term demand. Studies have shown that including meteorological and environmental factors can lead to better prediction accuracy for fitted models; however, the practical application of each factor needs to be evaluated individually to ensure their accuracy in predicting healthcare demand.

The lack of studies addressing the effects of meteorological and environmental factors on insect-and-mite-related dermatitis has motivated our multidisciplinary approach to investigating the following research questions: *(1) What are the impacts of factors, including weather conditions and air pollutants, on outpatient visits for this diagnosis? (2) To what extent can the studied factors improve the accuracy of demand prediction in clinical practice? (3) How do the impacts and prediction accuracies vary across subgroups defined by gender, age, disease type, and season?* For this purpose, we collect and analyze six years of data on papular urticaria and scabies cases among outpatients in Guangzhou, China. To identify the factors with the greatest impact on daily visits regarding outpatients with dermatitis, we apply a three-step framework: (1) We employ hierarchical cluster models based on Pearson correlation to reduce multicollinearity among candidate factors. (2) We utilize linear models to identify statistically significant factors in training data. (3) We rely on permutation importance to evaluate the predictive ability of each factor in test data. Our analysis also involves the examination of subgroups based on gender, age, disease type, and season to ensure the robustness of our findings. Ultimately, our results and conclusions can be applied to hospital operations with practical value for dealing with short-term fluctuations in demand.

## Materials and methods

### Outpatient data

We collect the visiting records of insect-and-mite-caused dermatitis outpatients from the Dermatology Hospital of Southern Medical University, which is the largest specialist hospital and the tertiary care center for dermatology in Guangzhou, China [40]. The city of Guangzhou is one of the largest cities in southern China with a population of 18.8 million and a typical subtropical humid-monsoon climate, making that papular urticaria and scabies common skin diseases. The study period ranges from the 1st of January 2015 to the 31st of December 2020 with a total of 2,156 days. The obtained 43,101 visiting entries are comprised of visiting dates and patients’ demographic information, including individual identification, gender, age, and diagnosed disease code. Repeated visiting records for the same patient within the same date are considered as a single entry [41]. The daily visit is referred to as the number of outpatients enrolled on the visiting entries each day. We utilize the International Classification of Diseases, 10th Revision (ICD-10) to identify the visiting entries diagnosed as papular urticaria (ICD-10: L28.2) or scabies (ICD-10: B86).

To account for calendar-related effects, we supplement the weekdays and the public holidays as the explanatory calendar variables. To capture the long-term increasing effects caused by the development of social economics, we supplement the linear trend of daily visits as the explanatory trend variable [13]. The descriptive statistics of daily visits are described in Table 1.

**Table 1:** Descriptive statistics of daily visits

*N* = 43,101	**All seasons**		**Spring**		**Summer**		**Fall**		**Winter**	
	Mean ± SD	Min - Max	Mean ± SD	Min - Max	Mean ± SD	Min - Max	Mean ± SD	Min - Max	Mean ± SD	Min - Max
**Total visits**	20.0 ± 9.6	1—62	22.7 ± 10.6	2—62	26.7 ± 7.8	8—57	18.3 ± 6.0	4—36	11.5 ± 5.5	1—32
**Visits by gender**										
Male	10.1 ± 4.9	1—30	11.0 ± 5.4	1—30	12.9 ± 4.5	3—29	9.6 ± 3.7	1—23	6.6 ± 3.4	1—20
Female	10.0 ± 5.7	1—34	11.8 ± 6.2	1—32	13.8 ± 5.0	2—34	8.7 ± 3.8	1—22	5.1 ± 3.1	1—17
**Visits by age**										
Age1 (< 18)	6.2 ± 3.7	1—36	6.9 ± 3.8	1—21	8.0 ± 4.0	1—36	5.3 ± 2.8	1—18	4.2 ± 2.6	1—16
Age2 (18—44)	9.8 ± 5.3	1—32	11.4 ± 6.0	1—32	13.0 ± 4.7	2—32	9.1 ± 3.7	2—21	5.4 ± 2.9	1—17
Age3 (45—59)	2.9 ± 1.8	1—12	3.2 ± 1.9	1—10	3.7 ± 1.9	1—12	2.6 ± 1.5	1—8	1.8 ± 1.0	1—6
Age4 (≥ 60)	2.3 ± 1.4	1—14	2.4 ± 1.5	1—14	2.6 ± 1.5	1—9	2.1 ± 1.3	1—10	1.8 ± 1.1	1—9
**Visits by disease**										
Papular urticaria	15.2 ± 8.9	1—54	18.1 ± 9.9	1—54	21.8 ± 7.0	6—49	13.1 ± 5.0	2—28	6.9 ± 4.0	1—24
Scabies	5.0 ± 2.7	1—19	4.8 ± 2.5	1—17	5.0 ± 2.6	1—14	5.3 ± 2.8	1—14	4.9 ± 2.9	1—19

*Mean* mean value, *SD* standard deviation, *Min* minimum value, *Max* maximum value

### Meteorological and environmental data

Meteorological and environmental data is collected based on the visiting date in our study period. For meteorological factors, daily values of ambient temperature, atmospheric pressure, relative humidity, wind speed, precipitation, evaporation, and sunshine duration are provided by China Meteorological Data Service Center [42]. For environmental air pollutants, daily concentrations of particulate matter (${{\text{PM}}}_{2.5}$ and ${{\text{PM}}}_{10}$), ozone (${{\text{O}}}_{3}$), nitrogen dioxide (${{\text{NO}}}_{2}$), and sulfur dioxide (${{\text{SO}}}_{2}$) are provided by Guangzhou Environmental Monitoring Center, which calculates the average observation values collected by 20 monitoring sites in our study area. The summary of meteorological and environmental data is described in Tables 2 and 3.

**Table 2:** Description (Mean and SD) of meteorological factors and air pollutants in Guangzhou, China, 2015—2020

*N* = 2,156	**All seasons**		**Spring**		**Summer**		**Fall**		**Winter**	
	Mean	SD	Mean	SD	Mean	SD	Mean	SD	Mean	SD
**Meteorological factors**										
Temperature (°C)	22.4	5.9	22.2	4.3	28.3	1.6	23.6	3.8	14.9	3.7
Pressure (hPa)	1004.9	6.7	1004.8	4.3	997.3	3.1	1005.6	4.7	1012.6	3.9
Humidity (%)	80.5	10.2	83.8	8.4	83.0	7.7	79.4	10.2	75.5	12.3
Wind speed (m/s)	2.3	1.0	2.2	0.9	2.1	0.8	2.3	1.0	2.6	1.1
Precipitation (mm)	6.2	17.3	8.2	20.6	10.9	22.5	3.4	10.1	2.1	9.7
Evaporation (mm)	3.6	1.9	3.2	2.2	4.1	1.9	4.0	1.8	3.0	1.5
Sunshine (hours)	4.4	3.8	2.7	3.5	5.4	3.5	5.6	3.7	3.8	3.7
**Air pollutants**										
${{\text{PM}}}_{2.5}$($\mathrm{\mu g}/{{\text{m}}}^{3}$)	32.5	18.6	31.6	15.8	22.0	10.5	34.4	15.0	43.0	24.5
${{\text{PM}}}_{10}$($\mathrm{\mu g}/{{\text{m}}}^{3}$)	53.9	26.7	52.6	22.7	39.3	14.3	58.5	24.2	66.1	34.8
${{\text{O}}}_{3}$($\mathrm{\mu g}/{{\text{m}}}^{3}$)	50.5	26.3	46.5	26.2	54.1	24.8	60.6	28.7	40.3	20.0
${{\text{SO}}}_{2}$($\mathrm{\mu g}/{{\text{m}}}^{3}$)	10.0	4.2	10.3	4.3	9.0	3.2	10.1	3.4	10.8	5.5
${{\text{NO}}}_{2}$($\mathrm{\mu g}/{{\text{m}}}^{3}$)	44.7	18.4	47.3	15.1	34.5	9.3	44.1	15.5	53.4	25.5

*Mean* mean value, *SD* standard deviation

**Table 3:** Description (Min and Max) of meteorological factors and air pollutants in Guangzhou, China, 2015—2020

*N* = 2,156	**All seasons**		**Spring**		**Summer**		**Fall**		**Winter**	
	Min	Max	Min	Max	Min	Max	Min	Max	Min	Max
**Meteorological factors**										
Temperature (°C)	3.4	32.2	8.4	30.9	23.6	32.2	11.4	30.5	3.4	23.7
Pressure (hPa)	985.7	1027.7	995.0	1016.8	985.7	1003.5	986.7	1015.9	1003.1	1027.7
Humidity (%)	31.0	100.0	51.0	100.0	64.0	99.0	36.0	99.0	31.0	98.0
Wind speed (m/s)	0.7	8.8	0.7	6.1	0.7	6.7	0.7	8.7	0.8	8.8
Precipitation (mm)	0.0	222.1	0.0	219.3	0.0	222.1	0.0	85.8	0.0	120.7
Evaporation (mm)	0.2	13.5	0.2	13.5	0.2	10.4	0.5	10.7	0.4	8.2
Sunshine (hours)	0.0	12	0.0	12.0	0.0	12.0	0.0	11.4	0.0	10.5
**Air pollutants**										
${{\text{PM}}}_{2.5}$($\mathrm{\mu g}/{{\text{m}}}^{3}$)	3.5	150.8	5.6	111.0	5.6	72.3	7.5	89.3	3.5	150.8
${{\text{PM}}}_{10}$($\mathrm{\mu g}/{{\text{m}}}^{3}$)	5.5	208.7	11.5	134.1	12.0	101.2	15.8	145.5	5.5	208.7
${{\text{O}}}_{3}$($\mathrm{\mu g}/{{\text{m}}}^{3}$)	3.7	189.0	5.1	139.7	6.7	189.0	3.7	138.8	4.2	94.6
${{\text{SO}}}_{2}$($\mathrm{\mu g}/{{\text{m}}}^{3}$)	3.9	38.1	4.1	27.1	4.1	21.5	3.9	28.2	4.0	38.1
${{\text{NO}}}_{2}$($\mathrm{\mu g}/{{\text{m}}}^{3}$)	8.3	173.2	17.2	95.2	15.2	64.6	8.3	113.8	9.0	173.2

*Min* minimum value, *Max* maximum value

### Statistical methods

We employ a three-step statistical framework, which includes variable selection, linear regression, and predictive evaluation, to investigate the correlation between daily visits and candidate explanatory variables. Consistent with prior studies, we account for the delayed impacts of several variables in the following manner. Firstly, considering the high autocorrelation of daily visits in the time series, we take 1 to 9 days lagged daily visits as candidate explanatory variables, represented by Visit_$i$ in our model [22]. Secondly, as exposure to high temperatures can have short-term effects, we also incorporate the daily temperature and its lags from 1 to 3 days as candidate explanatory variables in our model, denoted as Temperature_$i$ [22, 43].

We split the data into a training dataset and a test dataset. The training dataset comprises the initial five years of the data, covering a total of 1791 days from 2015 to 2019, while the test dataset includes the final year of our data, comprising 356 days in 2020. We perform the initial step of variable selection and the subsequent step of linear regression on the training dataset. In contrast, the test dataset is used for the final step of predictive evaluation.

#### First step: variable selection

**Fig. 1:**
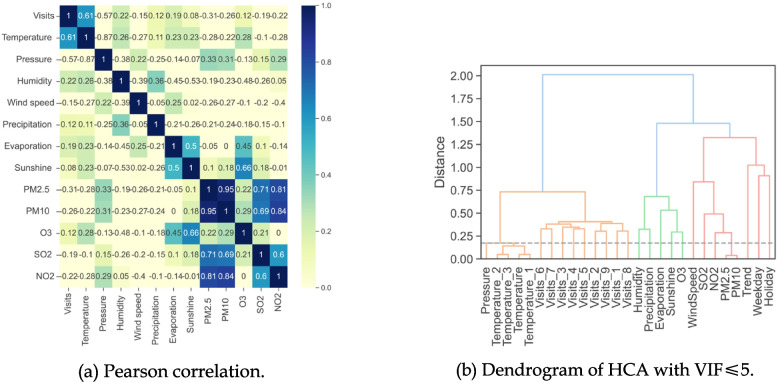
Pearson correlations and HCA of the main variables

In this step, we use hierarchical cluster analysis (HCA) to alleviate multicollinearity among candidate explanatory variables [44–46]. Multicollinearity can compromise our estimation of regression coefficients and evaluation of predictive ability. Figure 1(a) shows that multicollinearity exists in our candidate explanatory variables. By applying a distance measure like Pearson correlation, the HCA procedure sorts the candidate explanatory variables into several homogeneous clusters. Variables within a cluster exhibit high correlation, while those in different clusters show low correlation. For each cluster, we select one variable with the highest Pearson correlation with daily visits as the explanatory variable in the regression and prediction models.

We use the variance inflation factor (VIF) analysis to determine the number of clusters in HCA, while balancing the tradeoff between the explanatory variables and multicollinearity [47, 48]. We select the maximum number of clusters using a gray dashed line in Fig. 1(b) under the restriction that the VIF of the chosen explanatory variables is less than or equal to a prescribed threshold, $\delta$. In our study, we set $\delta =5$ to ensure that the final explanatory variables have a VIF of 5 or lower.

#### Second step: linear regression

In this step, we perform ordinary least squares (OLS) models to examine the relationship between daily visits and the explanatory variables selected in the first step. Similar to previous research [22, 49], we conduct separated OLS models for different seasons because many allergic diseases, like asthma and dermatitis, exhibit seasonal patterns due to allergens such as pollen, insects, and mites [50, 51]. Additionally, we also perform subgroup analyses for age (Age1, Age2, Age3, Age4), gender (male, female), and disease (papular urticaria, scabies), in correspondence with Yoo et al. [52]. We also assess the independence of error terms in the OLS models using the Ljung-Box Q test. The results show no autocorrelation in error terms for lags from 1 to 9 days, with a significance level of 0.05.

#### Third step: predictive evaluation

In this step, we conduct Permutation Importance Analysis (PIA) to assess the predictive abilities of explanatory variables in the trained Ordinary Least Squares (OLS) models [49]. Despite the explanatory variables having significant influence, they may still fail to predict daily visits because the in-sample performance of the trained models may differ from the out-of-sample performance in practical utilization [35, 36].

We adopt the mean square error (MSE) as the OLS model’s evaluation score. The permutation importance is defined as the improvement of MSE on the test dataset by comparing it with the MSE on a randomly shuffled dataset. Denote by $MS{E}_{\mathcal{D}}$ the MSE on the test dataset $\mathcal{D}$. For each variable $i$ in $\mathcal{D}$, we randomly shuffle the values of variable $i$ to generate a corrupted dataset ${\widetilde{\mathcal{D}}}_{i,k}$. The mathematical equation of permutation importance for variable $i$ in $\mathcal{D}$ is expressed as follows.

$$P{I}_{i}=\frac{MS{E}_{{\widetilde{\mathcal{D}}}_{i}}-MS{E}_{\mathcal{D}}}{MS{E}_{\mathcal{D}}}*100.$$

$P{I}_{i}$ reflects the increment of $MS{E}_{{\widetilde{\mathcal{D}}}_{i}}$ in percentage by comparing it with $MS{E}_{\mathcal{D}}$. A large positive $P{I}_{i}$ represents that randomly shuffling the value of variable $i$ can increase the MSE on the test dataset, meaning that variable $i$ is more important for the trained model from the perspective of predictive ability.

**Fig. 2:**
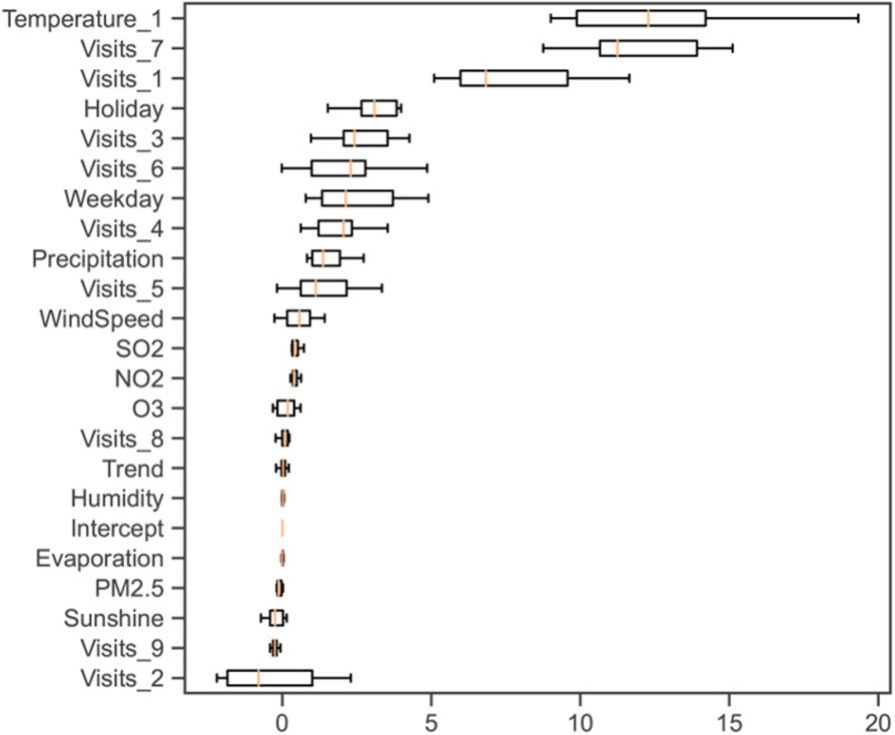
Permutation importance for total patients

Following Gao et al. [53], we repeat the above procedure 10 times to obtain 10 samples of $P{I}_{i}$. As shown in Fig. 2, we use boxplots to depict the distributions of $P{I}_{i}$. The yellow lines in the boxes represent the median values of $P{I}_{i}$, while the top and bottom of the boxes represent the first quartile and third quartile of $P{I}_{i}$, respectively. The variables are ranked and presented by the median values of $P{I}_{i}$ in decreasing order.

## Results

### Descriptive analysis

From January 1st, 2015 to December 31st, 2020, we obtain 43,101 individual records within a total of 2,156 days. Table 1 presents the mean, standard deviation (SD), minimum and maximum values of daily visits in our study. During the study period, the mean and SD values of daily outpatients are 20.0 and 9.6, respectively. The gender distribution of outpatients shows that males constitute 50.5% of the total outpatients, with a mean value of 10.1. Patients aged between < 18 and 18–44 years are the most frequently presenting age subgroups. These two age subgroups have mean values of 6.2 and 9.8, respectively. Notably, the mean number of papular urticaria outpatients is 15.2, which is three times higher than that of scabies outpatients. Seasonal patterns are observed, with summer having the highest mean value of 26.7 while winter having the lowest mean value of 11.5. Additionally, spring showed the highest fluctuation in total daily visits, as its SD value is the highest among all seasons.

Table 2 demonstrates the mean and SD values of meteorological factors and environmental air pollutants in our study. The mean values of ambient temperature, atmospheric pressure, relative humidity, wind speed, precipitation, evaporation, and sunshine duration are 22.4 °C, 1004.9 hPa, 80.5%, 2.3 m/s, 6.2 mm, 3.6 mm, and 4.4 h, respectively. Among the four seasons, spring shows the highest fluctuation of ambient temperature due to the highest SD value of 4.3. The mean values of ${{\text{PM}}}_{2.5}$, ${{\text{PM}}}_{10}$, ${{\text{O}}}_{3}$, ${{\text{SO}}}_{2}$, and ${{\text{NO}}}_{2}$ are 32.5 $\mathrm{\mu g}/{{\text{m}}}^{3}$, 53.9 $\mathrm{\mu g}/{{\text{m}}}^{3}$, 50.5 $\mathrm{\mu g}/{{\text{m}}}^{3}$, 10.0 $\mathrm{\mu g}/{{\text{m}}}^{3}$, and 44.7 $\mathrm{\mu g}/{{\text{m}}}^{3}$, respectively. Among the four seasons, winter shows the highest fluctuation in air quality, as the air pollutants in winter have the highest SD values in general.

Figure 1(a) shows the Pearson correlations among meteorological factors and environmental air pollutants during our study period. As can be observed, ${{\text{PM}}}_{2.5}$, ${{\text{PM}}}_{10}$, and ${{\text{NO}}}_{2}$ are highly correlated because the Pearson correlations are higher than 0.8. Similarly, a relatively high correlation between sunshine duration and ${{\text{O}}}_{3}$ is also identified as the Pearson correlation is higher than 0.6. Due to the limitation of picture size, the Pearson correlations among lagged variables, which are also highly correlated, are not illustrated. For example, the Pearson correlations among lagged temperatures are higher than 0.8.

Figure 1(b) exhibits the dendrogram of HCA for all candidate explanatory variables with $\delta =5$. The gray dashed line shows how these variables are clustered. Temperature and its lagged variables are classified into the same cluster, with Temperature_1 being selected as the explanatory variable. Likewise, ${{\text{PM}}}_{2.5}$ and ${{\text{PM}}}_{10}$ are also classified into another cluster, with ${{\text{PM}}}_{2.5}$ being selected as the explanatory variable. Each remaining variable is considered as a single cluster and, hence, the explanatory variable. Finally, the VIF of selected explanatory variables is 4.9, which is guaranteed by the threshold $\delta$.

### Daily outpatient visits for total patients

The OLS results for total patients are summarized in Table 4. The adjusted ${{\text{R}}}^{2}$ value is 0.626, indicating most daily visits can be explained by the selected explanatory variables. The lagged daily visits, except for lags of 8 and 9 days, show positive effects at the 1% significance level. The one-day lagged temperature is selected as the explanatory variable, which has positive effects with the significance level at 1%. The daily visit increases linearly over the one-day lagged temperature on average by 0.283. Except for the one-day lagged temperature, only precipitation takes negative effects with the significance level at 1%. There is no strong evidence suggesting that daily visits can be affected by air pollutants, as the regression coefficients of air pollutants are not statistically significant at levels less than 10%. However, the calendar effects and trend effects are identified. The daily visits are associated with a sharp increase of 2.464 on weekdays, but a sharp decrease of 2.030 on holidays. The increasing trend of daily visits implies that the demands may increase naturally due to the development of social economics.

**Table 4:** Factors associated with daily outpatient visits for total patients (OLS models)

	Total (*N* = 1,791)	
	Coef	SE
Visit_1	0.134***	0.023
Visit_2	0.082***	0.024
Visit_3	0.075***	0.023
Visit_4	0.086***	0.023
Visit_5	0.107***	0.024
Visit_6	0.116***	0.023
Visit_7	0.182***	0.024
Visit_8	-0.009	0.023
Visit_9	0.011	0.023
Temperature_1	0.283***	0.042
Humidity	-0.001	0.025
Wind speed	-0.263	0.198
Sunshine	-0.072	0.056
Precipitation	-0.026***	0.009
Evaporation	-0.010	0.097
${{\text{PM}}}_{2.5}$	-0.006	0.016
${{\text{O}}}_{3}$	-0.010	0.009
${{\text{SO}}}_{2}$	0.061	0.063
${{\text{NO}}}_{2}$	0.006	0.017
Weekdays	2.464***	0.332
Holidays	-2.030***	0.588
Trend	0.001**	0.000
Adjusted ${{\text{R}}}^{2}$	0.626	

*SE* represents the standard error of coefficients

^***^ $$p<0.01$$, **$$p<0.05$$, *$$p<0.1$$

Moreover, the out-of-sample performance of the OLS model is illustrated in Fig. 2. Temperature-related variables are demonstrated to be predictive, due to high values of permutation importance. The one-day lagged temperature ranks first with the median value of permutation importance being 12.3, indicating that the predictive ability of the OLS model is improved by 12.3% by this variable. The daily visits at the lags of 7 and 1 days rank second and third with the median values of permutation importance being 11.2 and 6.8, respectively, implying that the predictive ability of the OLS model is also greatly improved by these two variables. Besides, holidays and weekdays can slightly improve the prediction accuracy with the median values of permutation importance being 3.1 and 2.1, respectively. Such results are consistent with the OLS model, as the regression coefficients of these mentioned variables are statistically significant at the level of 1%.

However, significant levels do not necessarily represent high predictive abilities (e.g., high values of permutation importance). Specifically, even though the trend has positive effects with the significance level at 5%, it fails to predict daily visits as its median value of permutation importance is only 0.1, which is less than that of some other nonsignificant variables, such as wind speed and ${{\text{SO}}}_{2}$. Besides, the daily visit at the lag of 2 days holds the last rank but still has a significant regression coefficient with the significance level at 1%, indicating that its predictive ability in the OLS model is unreliable.

### Daily outpatient visits by seasons

The OLS results for subgroups by seasons are summarized in Table A1. The adjusted ${{\text{R}}}^{2}$ values are 0.654, 0.169, 0.300, and 0.325 for spring, summer, fall, and winter, respectively. Such results show that daily visits in spring can be better explained by the OLS model. Many lagged daily visits are reported to be positively significant in summer and winter. However, the daily visit at the lag of 8 days is negatively significant in spring with the significance level at 5%; the daily visit at the lag of 3 days is negatively significant in fall with the significance level at 10%. Besides, the daily visit at the lag of 9 days is not significant in all seasons.

The impacts of temperatures are robust but with various intensities among season subgroups. The daily visit increases linearly over the daily temperature on average by 0.860 in spring, and over the one-day lagged temperature on average by 0.409 and 0.260 in fall and winter, respectively. However, the daily visit decreases linearly over the three-day lagged temperature on average by 0.714 in summer. This opposite effect can be explained by the adverse effect of high temperatures on the activity of insects and mites. The influence of other weather factors is not robust among seasons. Wind speed is significantly associated with fewer daily visits in fall and winter. Sunshine duration has a positive correlation in fall but a negative correlation in spring with daily visits. No other weather factors are found to be significantly correlated with daily visits. Compared with weather factors, air pollutants are less influential in daily visits except for ${{\text{O}}}_{3}$ and ${{\text{NO}}}_{2}$. Specifically, ${{\text{O}}}_{3}$ is linked to more visits in spring but with only a significance level at 10%. A unit increase in ${{\text{NO}}}_{2}$ is associated with 0.106 and 0.070 fewer daily visits in spring and fall at the significance levels of 5% and 1%, respectively. The calendar-related factors are found to be effective in many cases. The outpatient department is linked to more visits on weekdays for all seasons, while fewer visits on holidays for spring and fall. A significantly increasing trend of daily visits is identified in all seasons except for summer.

**Fig. 3:**
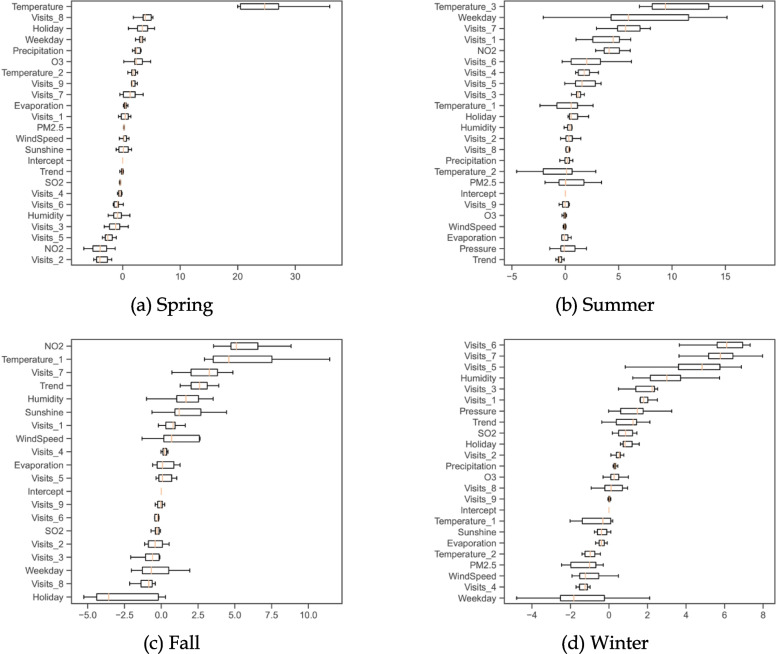
Permutation importance for subgroups by seasons

Moreover, the out-of-sample performance of OLS models with respect to seasons is illustrated in Fig. 3. It confirms that temperature-related variables are identified to be predictive, based on high values of permutation importance. The daily temperature and three-day lagged temperature rank first in spring and summer with the median values of permutation importance being 24.7 and 9.4, respectively. The one-day lagged temperature ranks second in fall with the median value of permutation importance being 4.6. However, we find that the one-day lagged temperature fails to predict daily visits in winter. Even though the one-day lagged temperature has positive effects at the significance level of 5%, its median value of permutation importance is only $-0.3$, implying that including the one-day lagged temperature in the OLS model does not help improve the prediction accuracy. Among all air pollutants, ${{\text{NO}}}_{2}$ has the highest rank in fall with the median value of permutation importance being 5.1. Other explanatory variables with significance levels of 1% (mainly lagged daily visits, weekdays, holidays, and the trend) report the median values of permutation importance less than 5.

### Daily outpatient visits by age

The OLS regression results for subgroups by age are summarized in Table A2. The adjusted ${{\text{R}}}^{2}$ values are 0.338, 0.548, 0.210 and 0.110 for Age1, Age2, Age3 and Age4, respectively. Such results show that daily visits from Age2 can be better explained by the OLS model. Most lagged daily visits are positively related to daily visits with many of them being significant. Particularly, the daily visit at the lag of 6 days is positively significant for all age subgroups at the significance level of 1%.

The impacts of temperatures are robust but with various intensities among age subgroups. The one-day lagged temperature is more influential for Age1 and Age2 with the linearly increasing rates of 0.080 and 0.136, respectively, while the daily temperature is more effective for Age3 and Age4 with the linearly increasing rates of 0.059 and 0.050, respectively. Most other weather factors cannot significantly affect daily visits. Only sunshine duration is associated with fewer daily visits in Age1 and Age3, and precipitation in Age2. By contrast, there is no significant evidence suggesting that daily visits can be affected by air pollutants. Besides, the effects of weekdays hold at the significance level of 1% for all age subgroups. The daily visit increases by 1.509, 0.694 and 0.716 on weekdays for Age2, Age3 and Age4, respectively. Conversely, the daily visit decreases by 0.630 on weekdays for Age1. The effects of holidays hold at the significance level of 1% only for Age2 and Age4. An increasing trend of daily visits is confirmed for all age subgroups except for Age3.

**Fig. 4:**
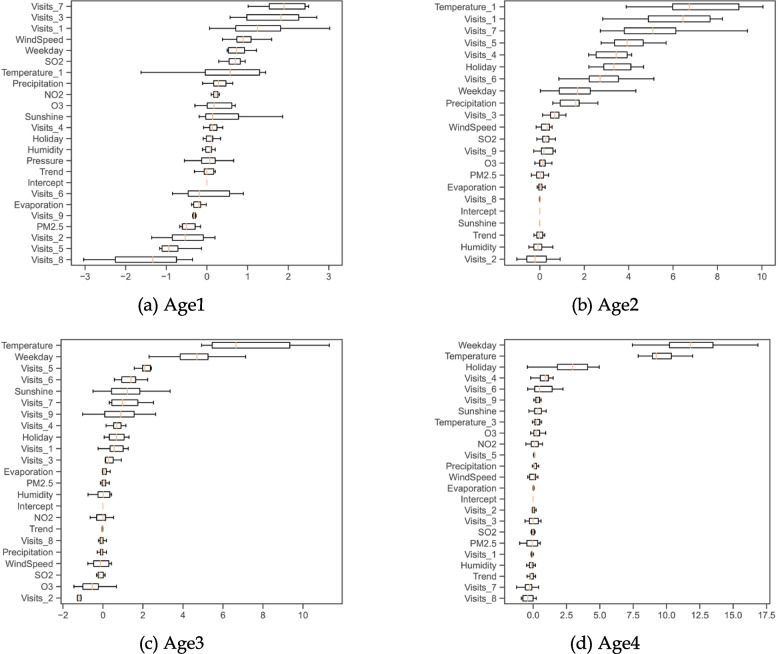
Permutation importance based on MSE by subgroups of age

Moreover, the out-of-sample performance of OLS models with respect to age groups is illustrated in Fig. 4. It demonstrates that temperature-related variables are identified to be predictive, based on high values of permutation importance. The one-day lagged temperature ranks first in Age2 with the median permutation importance of 6.7. The daily temperature ranks first and second in Age3 and Age4 with the median permutation importance being 6.6 and 9.2, respectively. However, we find that the one-day lagged temperature fails to predict daily visits in Age1. Although the one-day lagged temperature has positive effects at the significance level of 1%, its median value of permutation importance is only 0.6, implying that including the one-day lagged temperature in the OLS model can only reduce the MSE by 0.6%. Other highly ranked explanatory variables (e.g., variables with the first three ranks), including lagged daily visits, weekdays and holidays, have significance levels of 1%. Air pollutants do not play a vital role in predicting daily visits, as their median values of permutation importance are very small (e.g., less than 1).

### Daily outpatient visits by gender

The OLS results for subgroups by gender are summarized in Table A3. The adjusted ${{\text{R}}}^{2}$ values are 0.427 and 0.565 for males and females, respectively. Such results suggest that daily visits of female patients can be better explained by the OLS model. The effects of lagged daily visits on males and females are quite similar. For example, the daily visits at the lags of 2, 3, 6 and 7 days show positive effects for both males and females at the significance level of 1%.

The impacts of temperatures are robust but with various intensities among gender subgroups. The daily visit increases linearly over the one-day lagged temperature on average by 0.178 and 0.150 for males and females, respectively. Wind speed and precipitation are negatively associated with the daily visits for males and females, respectively, at the significance level of 5%. No other weather factors are found to be significantly correlated with daily visits. By contrast, there is no significant evidence suggesting that daily visits can be affected by air pollutants. Besides, females are more likely to be affected by calendar-related factors. The outpatient department is linked to 0.808 and 1.582 more visits on weekdays but 0.628 and 1.546 fewer visits on holidays for males and females, respectively. Increasing trends of daily visits for both males and females are confirmed under the significance level of less than 5%.

**Fig. 5:**
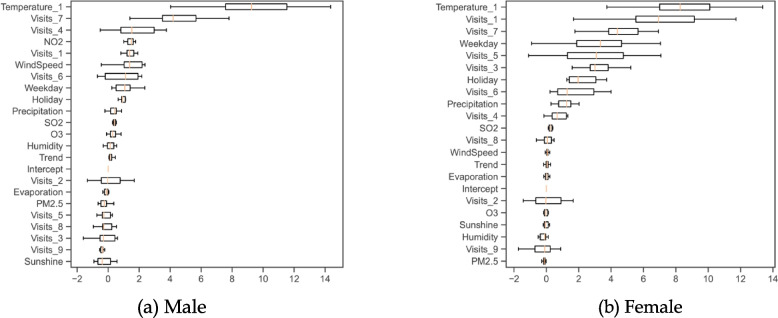
Permutation importance for subgroups by gender

Moreover, the out-of-sample performance of OLS models with respect to gender subgroups is illustrated Fig. 5. It reveals that temperature-related variables are identified to be predictive, as the one-day lagged temperature ranks first with the median values of permutation importance being 9.3 and 8.3 for both males and females, respectively. Consistent with the regression results, the air pollutants do not play a vital role in predicting daily visits, as their median values of permutation importance are very small (e.g., mostly less than 1). Other explanatory variables with significant levels less than 5% have small median values of permutation importance (e.g., all less than 5). Particularly, even though the trend has positive effects with the significance level at 5% for males or females, its median values of permutation importance are less than 1, which is less than that of some other nonsignificant variables, such as ${{\text{SO}}}_{2}$.

### Daily outpatient visits by disease

The OLS results for subgroups by disease are summarized in Table A4. The adjusted ${{\text{R}}}^{2}$ values are 0.700 and 0.088 for papular urticaria and scabies, respectively. Such results suggest that daily visits of papular urticaria patients can be better explained by the OLS model. The lagged daily visits are more effective for papular urticaria patients than scabies patients. Considering the significance level at 1%, the daily visits at the lags from 1 to 7 are positively significant for papular urticaria patients, but only the daily visits at the lags of 6 and 7 are positively significant for scabies patients.

The impacts of temperatures are inconsistent in disease subgroups. The daily visit increases linearly over the one-day lagged temperature on average by 0.215 with the significance level at 1% for papular urticaria patients. However, the daily visit decreases linearly over the three-day lagged temperature on average by 0.031 for scabies patients with the significance level at 10%. As for other weather factors, precipitation links to fewer papular urticaria patients, while evaporation inclines to more scabies patients. Among air pollutants, only ${{\text{SO}}}_{2}$ reports a positive association with more scabies patients at the significance level of 1%. Besides, papular urticaria patients are more likely to be affected by calendar-related factors. The outpatient department is linked to 1.801 and 0.661 more visits on weekdays but 1.323 and 0.716 fewer visits on holidays for papular urticaria and scabies patients, respectively. Compared with papular urticaria patients, scabies patients have a higher increasing rate of daily visits at the significance level of 1%.

**Fig. 6:**
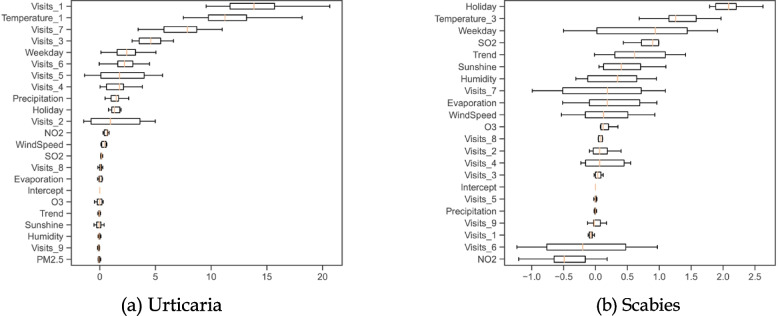
Permutation importance for subgroups by disease

Moreover, the out-of-sample performance of OLS models with respect to disease subgroups is illustrated in Fig. 6. It shows that temperature-related variables exhibit leading abilities in prediction accuracy. As observed, the one-day lagged temperature ranks second with the median values of permutation importance being 11.2 for papular urticaria patients. Similarly, the three-day lagged temperature ranks second with the median values of permutation importance being 2.2 for scabies patients. Besides, the daily visits at the lags of 1 and 9 also have relatively high median values of permutation importance for papular urticaria patients (e.g., all greater than 5). All explanatory variables have low median values of permutation importance for scabies patients (e.g., all less than 5). As in the previous analyses, explanatory variables with significance levels below 5% can also have low values of permutation importance. As for papular urticaria patients, wind speed is significant at the level of 5% but its median value of permutation importance is only 0.4, implying that wind speed is not a reliable predictor. It is noteworthy to see that all significant explanatory variables for scabies patients have very limited predictive abilities due to low median values of permutation importance.

## Discussion

This study aims to investigate the effects of weather and air pollution on the number of outpatient visits for dermatitis caused by insects and mites at the Guangdong Provincial Dermatology Hospital over six years. Prior research has demonstrated the influence of weather and air pollution on medical demands for different illnesses [17, 20, 25]. However, there is a lack of studies investigating dermatitis resulting from insects and mites. Furthermore, the predictive capacity of these factors regarding the number of outpatient visits has been understudied, resulting in limited understanding.

### The influence of temperatures

Our results find that short-term high temperatures are positively correlated with an increase in daily visits from dermatitis patients. This correlation is consistent with theoretical support presented in previous literature [54], as high temperatures increase the probability of pruritus, a common symptom of papular urticaria, and can exacerbate the condition due to persistent scratching. Additionally, several recent publications have empirically investigated the link between temperatures and skin dryness. Findings indicate that as temperatures rise, skin dryness may occur due to the direct effect on thermoregulatory vasculature, and indirectly through neural mechanisms that affect skin temperature [55].

The explanations of temperature influence are summarized as follows. Firstly, insect bites are typically considered the primary cause of papular urticaria [56]. Patients experience a higher incidence of this condition in the spring, as indicated by the significantly positive association and high permutation importance. During the spring season, as temperatures rise and the climate changes, insects become more active, leading to a higher likelihood of developing papular urticaria as a result of insect bites. Secondly, unlike papular urticaria, which stems from outdoor insect bites, scabies is typically contracted through indoor sources, for instance, direct skin-to-skin contact or contact with contaminated objects such as bedding, clothing, and towels [57]. As outdoor temperatures decrease, individuals tend to stay indoors, increasing their likelihood of acquiring scabies. Our research further reveals that short-term temperatures have a strong ability to predict outpatient visits, which is a new finding in this field.

Additionally, our findings indicate that one-day lagged and daily temperatures have distinct impacts on different age groups. Specifically, we find that populations under the age of 45 are more affected by the one-day lagged temperature, whereas the remaining populations are more affected by the daily temperature. These results are in line with previous studies but with some differences. For example, Nie et al. [57] show that the one-day lagged temperature has a greater impact on young and middle-aged urticaria patients, whereas Narla et al. [58] show that the three-day lagged temperature has a significant impact on all urticaria patients.

### The influence of other factors

Except for short-term temperatures, the other weather variables are not significant in our regression models. Previous research supports that exposure to sunshine is a significant risk factor for skin diseases [59]. It indicates that sunshine has a greater effect on middle-aged and elderly populations than other populations and has the greatest effect on outpatient services in the fall season. However, our results do not support this conclusion except for the fall subgroup. Extended exposure to ultraviolet radiation can cause wrinkles and aging signs by breaking down collagen fibers in the skin. Besides, previous research also supports that humidity has a significant negative impact on outpatient services for dermatology [22]. It suggests that the increase in dermatology patients during the warmer season can be attributed to the higher bacterial presence due to humid conditions, which increases the likelihood of getting bitten by mosquitoes and causes discomfort. However, our results do not support this conclusion. In addition, other meteorological variables such as wind speed, atmospheric pressure, and evaporation exhibit insignificance in our regression models. Possible reasons for these observations are as follows: (1) The primary etiology of papular urticaria is conventionally attributed to insect bites [56]. Elevated temperatures augment insect activity, thereby elevating the susceptibility to papular urticaria due to increased occurrences of insect bites. However, it is conceivable that wind speed, atmospheric pressure, and evaporation exert minimal influence on insect activity. (2) Elevated temperatures contribute to an increased likelihood of pruritus, a prevalent symptom of papular urticaria. Nevertheless, the influence of wind speed, atmospheric pressure, and evaporation on pruritus remains statistically insignificant.

With respect to air pollution, previous studies draw various conclusions. Abolhasani et al. [60] demonstrate that exposure to air pollution can exacerbate skin conditions, resulting in severe instances of papular urticaria. ${{\text{NO}}}_{2}$ is considered the most significant for outpatient services for skin disease patients in autumn. However, this study does not show a significant correlation between ${{\text{NO}}}_{2}$ concentration and the number of daily visits of dermatitis outpatients, nor does it provide conclusive evidence of the high predictive ability of ${{\text{NO}}}_{2}$ concentration. As mentioned before, the main causes of papular urticaria and scabies are insect bites and mite contact, which are thought to be less influenced by air pollutants. As verified by many publications [39], calendar effects exist in our study. Daily visits are expected to rise during weekdays, while they are prone to decrease during holidays. These impacts of weekdays and holidays are significant across most subgroups at the significance level below 5%. However, our study also reveals that calendar effects provide limited values in predicting daily visits. This conclusion contributes to the literature on calendar effects.

## Conclusions

Our results are consistent with existing theoretical assumptions, indicating that short-term high temperatures lead to an increase in daily visits. Additionally, our findings demonstrate that short-term high temperatures exhibit strong predictive capabilities. However, it is interesting to note that the increased significance level does not necessarily result in an improved predictive capacity. For instance, considering all patients, the daily visit at a lag of two days reports the smallest value of permutation importance (see Fig. 2 and Table A1). Nevertheless, this value remains statistically significant at the 1% level. It is noteworthy that there remains a lack of research examining the impact of weather conditions and air pollutants on the healthcare demand of insect-and-mite-caused dermatitis patients.

In terms of methodology, we employ a comprehensive integrated framework that combines the steps of variable selection, linear regression analysis and predictive evaluation. This approach enables us to analyze the statistical impacts of weather conditions and air pollutants on daily visits of outpatients with insect-and-mite-caused dermatitis, as well as to evaluate their predictive capacity. We present a detailed summary of the conclusions drawn from our results as follows.

Short-term temperatures are positively associated with daily visits, with the one-day lagged temperature having a significant impact on daily visits of total outpatients in the OLS model ($p<0.01$). However, this relationship does not hold for two subgroups: summer and scabies. In the summer subgroup, the three-day lagged temperature has a negative effect on daily visits with a significance level of 5%. This outcome may be explained by the adverse impact of high temperatures on insect activity. In the scabies subgroup, the three-day lagged temperature has a negative effect at a significance level of 10%. This result is attributed to the less significant influence of short-term temperature on the activities of scabies compared to that of insects. Furthermore, the effects of short-term temperature exhibit heterogeneity across subgroups, with patients appearing to be more sensitive to changes in spring than in other seasons. The rising temperature in spring stimulates insect activities, leading to an increased likelihood of dermatitis resulting from insect bites.

Short-term temperatures exhibit high abilities in predicting daily visits. As for the OLS model concerning total patients, the one-day lagged temperature has the highest median value of permutation importance. This finding aligns with empirical evidence revealing significant positive effects of one-day lagged temperature at a 1% significance level. Additionally, this conclusion is robust in most subgroups, as short-term temperatures rank among the top two variables in terms of permutation importance. However, the one-day lagged temperature inadequately predicts daily visits in subgroups of winter and Age1. The median value of permutation importance in winter is $-0.3$ and 0.6 in Age1, suggesting that short-term temperatures provide little improvement to the prediction accuracy.

Air pollution has insignificant impacts on daily visits, resulting in weak predictive abilities. This is intuitive since air pollution does not directly affect the activities of insects and scabies. The OLS model for total patients reveals two crucial findings. Firstly, none of the air pollutants show any correlations with daily visits below a significance level of 10%. Secondly, the air pollutants demonstrate weak predictive abilities over daily visits, with ${{\text{SO}}}_{2}$ having the highest median value of permutation importance (0.4). Our findings are robust in most subgroups except for the subgroup of fall. In this subgroup, ${{\text{NO}}}_{2}$ reveals not only a negative association with daily visits at the significance level of 1%, but also the strongest predictive ability with the highest median value of permutation importance.

Weekdays, holidays and trends have significant impacts on daily visits, but with weak predictive abilities. The OLS models for total patients reveal a positive association between weekdays and daily visits at a significance level of 1%. The opposite association holds between holidays and daily visits. However, their median values of permutation importance are relatively small, implying weak predictive abilities. Although our findings reveal an upward trend in daily visits, the trend factor is inadequate to predict daily visits in out-of-sample performance. With the exception of some subgroups, such as summer and Age3, our findings are robust.

### Supplementary Information

**Supplementary Material 1.**

## Data Availability

Daily meteorological data can download from the publicly accessible China Meteorological Data Service Centre (http://data.cma.cn). Daily data of air pollutants were collected from Guangzhou Municipal Ecological Environment Bureau (http://sthjj.gz.gov.cn). The datasets of insect-and-mite-caused dermatitis outpatients generated and analyzed during the current study are not publicly available due to the requirement of the Dermatology Hospital of Southern Medical University but are available from the corresponding author on reasonable request.

## Abbreviations

PM: Particulate matter
PM_10_: Particulate matter (particles of < 10 *µ*m in aerodynamic diameter)
PM_2.5_: Fine particulate matter (particles of < 2.5 *µ*m in aerodynamic diameter)
O_3_: Ozone
NO_2_: Nitrogen dioxide
SO_2_: Sulfur dioxide
MSE: Mean square error
