# Supplementary material for: Effects of weather and air pollution on outpatient visits for insect-and-mite-caused dermatitis: an empirical and predictive analysis
Source: BMC Public Health. 2024 Feb 28;24:633. doi: 10.1186/s12889-024-18067-y (PMC11218201; doi:10.1186/s12889-024-18067-y)
Supplement: Supplementary file 1 — Supplementary Material 1. [file 12889_2024_18067_MOESM1_ESM.docx]

# Appendix

## OLS results for subgroups

**Table A1** Factors associated with daily visits by season (OLS models)

|  | Total (N = 1,791) | | Spring (N = 451) | | Summer (N = 451) | | Fall (N = 446) | | Winter (N = 416) | |
| --- | --- | --- | --- | --- | --- | --- | --- | --- | --- | --- |
|  | Coef. | SE | Coef. | SE | Coef. | SE | Coef. | SE | Coef. | SE |
| Visit_1 | 0.134*** | 0.023 | 0.078 | 0.047 | 0.087* | 0.047 | 0.040 | 0.047 | 0.028 | 0.049 |
| Visit_2 | 0.082*** | 0.024 | 0.124*** | 0.047 | 0.035 | 0.047 | -0.040 | 0.046 | 0.009 | 0.049 |
| Visit_3 | 0.075*** | 0.023 | 0.139*** | 0.046 | 0.029 | 0.045 | -0.089* | 0.045 | 0.030 | 0.049 |
| Visit_4 | 0.086*** | 0.023 | 0.035 | 0.046 | 0.040 | 0.046 | 0.016 | 0.045 | -0.023 | 0.048 |
| Visit_5 | 0.107*** | 0.024 | 0.067 | 0.048 | 0.081* | 0.047 | 0.038 | 0.046 | 0.076 | 0.048 |
| Visit_6 | 0.116*** | 0.023 | 0.042 | 0.046 | 0.070 | 0.045 | 0.027 | 0.045 | 0.129*** | 0.048 |
| Visit_7 | 0.182*** | 0.024 | 0.157*** | 0.046 | 0.096** | 0.047 | 0.119** | 0.046 | 0.111** | 0.049 |
| Visit_8 | -0.009 | 0.023 | -0.098** | 0.045 | 0.016 | 0.046 | -0.057 | 0.046 | -0.032 | 0.048 |
| Visit_9 | 0.011 | 0.023 | -0.052 | 0.044 | -0.019 | 0.045 | -0.015 | 0.047 | -0.005 | 0.048 |
| Temperature |  |  | 0.860*** | 0.110 |  |  |  |  |  |  |
| Temperature_1 | 0.283*** | 0.042 |  |  | 0.344 | 0.322 | 0.409*** | 0.090 | 0.260** | 0.112 |
| Temperature_2 |  |  | -0.096 | 0.127 | 0.428 | 0.342 |  |  | 0.100 | 0.105 |
| Temperature_3 |  |  |  |  | -0.714** | 0.281 |  |  |  |  |
| Humidity | -0.001 | 0.025 | 0.093 | 0.067 | 0.022 | 0.089 | 0.058 | 0.041 | -0.044 | 0.034 |
| Wind speed | -0.263 | 0.198 | -0.438 | 0.478 | 0.044 | 0.540 | -0.741** | 0.367 | -0.477* | 0.261 |
| Sunshine | -0.072 | 0.056 | -0.246* | 0.142 |  |  | 0.215** | 0.094 | -0.045 | 0.079 |
| Pressure |  |  |  |  | 0.135 | 0.117 |  |  | -0.109 | 0.084 |
| Precipitation | -0.026*** | 0.009 | -0.025 | 0.019 | -0.013 | 0.017 |  |  | -0.025 | 0.022 |
| Evaporation | -0.010 | 0.097 | 0.178 | 0.195 | 0.176 | 0.227 | 0.158 | 0.179 | -0.091 | 0.199 |
| $\mathrm{PM}_{2.5}$ | -0.006 | 0.016 | 0.005 | 0.042 | -0.105 | 0.067 |  |  | -0.018 | 0.014 |
| $O_{3}$ | -0.010 | 0.009 | 0.040* | 0.021 | 0.002 | 0.023 |  |  | -0.011 | 0.019 |
| $SO_{2}$ | 0.061 | 0.063 | -0.045 | 0.155 |  |  | 0.080 | 0.120 | 0.049 | 0.074 |
| $\mathrm{NO}_{2}$ | 0.006 | 0.017 | -0.106** | 0.045 | 0.113* | 0.066 | -0.070*** | 0.027 |  |  |
| Weekdays | 2.464*** | 0.332 | 1.287* | 0.721 | 4.650*** | 0.812 | 1.745*** | 0.571 | 2.328*** | 0.486 |
| Holidays | -2.030*** | 0.588 | -4.056*** | 1.214 | -1.684 | 2.007 | -3.586*** | 0.871 | -0.481 | 0.989 |
| Trend | 0.001** | 0.000 | 0.013*** | 0.005 | 0.003 | 0.003 | 0.019*** | 0.003 | 0.012*** | 0.003 |
| Adjusted $R^{2}$ | 0.626 |  | 0.654 |  | 0.169 |  | 0.300 |  | 0.325 |  |

Note: *** $p<0.01$, ** $p<0.05$, * $p<0.1$; SE represents the standard error of coefficients.

**Table A2** Factors associated with daily visits by age (OLS models)

|  | Age1 (N = 1,764) | | Age2 (N = 1,780) | | Age3 (N = 1,525) | | Age4 (N = 1,333) | |
| --- | --- | --- | --- | --- | --- | --- | --- | --- |
|  | Coef. | SE | Coef. | SE | Coef. | SE | Coef. | SE |
| Visit_1 | 0.095*** | 0.024 | 0.163*** | 0.023 | 0.042* | 0.025 | -0.006 | 0.027 |
| Visit_2 | 0.048** | 0.024 | 0.070*** | 0.024 | 0.039 | 0.025 | -0.009 | 0.027 |
| Visit_3 | 0.097*** | 0.024 | 0.036 | 0.023 | 0.056** | 0.025 | 0.045* | 0.027 |
| Visit_4 | 0.015 | 0.024 | 0.085*** | 0.023 | 0.038 | 0.025 | 0.055** | 0.027 |
| Visit_5 | 0.037 | 0.024 | 0.143*** | 0.024 | 0.084*** | 0.025 | 0.003 | 0.027 |
| Visit_6 | 0.082*** | 0.024 | 0.121*** | 0.023 | 0.065*** | 0.025 | 0.090*** | 0.027 |
| Visit_7 | 0.162*** | 0.024 | 0.125*** | 0.024 | 0.074*** | 0.025 | 0.037 | 0.027 |
| Visit_8 | 0.086*** | 0.024 | -0.001 | 0.024 | 0.014 | 0.025 | 0.030 | 0.027 |
| Visit_9 | 0.018 | 0.024 | 0.028 | 0.023 | 0.067*** | 0.025 | 0.025 | 0.027 |
| Temperature |  |  |  |  | 0.059*** | 0.012 | 0.050*** | 0.013 |
| Temperature_1 | 0.080*** | 0.027 | 0.136*** | 0.024 |  |  |  |  |
| Temperature_3 |  |  |  |  |  |  | 0.007 | 0.013 |
| Humidity | -0.005 | 0.013 | 0.007 | 0.015 | 0.003 | 0.008 | -0.002 | 0.007 |
| Wind speed | -0.136 | 0.103 | -0.105 | 0.116 | -0.042 | 0.060 | 0.030 | 0.053 |
| Sunshine | -0.052* | 0.029 | -0.000 | 0.034 | -0.030* | 0.017 | -0.014 | 0.015 |
| Pressure | -0.032 | 0.022 |  |  |  |  |  |  |
| Precipitation | -0.007 | 0.005 | -0.014*** | 0.006 | -0.001 | 0.003 | 0.001 | 0.002 |
| Evaporation | 0.028 | 0.050 | -0.033 | 0.059 | 0.016 | 0.027 | -0.002 | 0.025 |
| $\mathrm{PM}_{2.5}$ | -0.004 | 0.008 | -0.008 | 0.008 | -0.001 | 0.005 | 0.004 | 0.004 |
| $O_{3}$ | -0.005 | 0.005 | -0.004 | 0.005 | 0.003 | 0.003 | -0.001 | 0.002 |
| $SO_{2}$ | 0.034 | 0.033 | 0.037 | 0.036 | -0.011 | 0.019 | 0.006 | 0.018 |
| $\mathrm{NO}_{2}$ | 0.001 | 0.009 |  |  | -0.002 | 0.005 | -0.003 | 0.005 |
| Weekdays | -0.630*** | 0.169 | 1.509*** | 0.201 | 0.694*** | 0.097 | 0.716*** | 0.093 |
| Holidays | 0.508* | 0.303 | -1.495*** | 0.357 | -0.287 | 0.182 | -0.652*** | 0.169 |
| Trend | 0.001*** | 0.000 | 0.000** | 0.000 | 0.000 | 0.000 | 0.000** | 0.000 |
| Adjusted $R^{2}$ | 0.338 |  | 0.548 |  | 0.210 |  | 0.110 |  |

Note: *** $p<0.01$, ** $p<0.05$, * $p<0.1$; SE represents the standard error of coefficients.

**Table A3** Factors associated with daily visits by gender (OLS models)

|  | Male (N = 1,788) | | Female (N = 1,784) | |
| --- | --- | --- | --- | --- |
|  | Coef. | SE | Coef. | SE |
| Visit_1 | 0.051** | 0.024 | 0.147*** | 0.023 |
| Visit_2 | 0.082*** | 0.024 | 0.071*** | 0.024 |
| Visit_3 | 0.076*** | 0.024 | 0.080*** | 0.023 |
| Visit_4 | 0.104*** | 0.023 | 0.037 | 0.023 |
| Visit_5 | 0.026 | 0.024 | 0.141*** | 0.024 |
| Visit_6 | 0.105*** | 0.023 | 0.085*** | 0.023 |
| Visit_7 | 0.159*** | 0.024 | 0.146*** | 0.024 |
| Visit_8 | 0.029 | 0.024 | 0.020 | 0.024 |
| Visit_9 | 0.013 | 0.024 | 0.051** | 0.023 |
| Temperature_1 | 0.178*** | 0.026 | 0.150*** | 0.026 |
| Humidity | -0.011 | 0.016 | 0.011 | 0.016 |
| Wind speed | -0.279** | 0.126 | -0.039 | 0.122 |
| Sunshine | -0.047 | 0.036 | -0.025 | 0.035 |
| Precipitation | -0.008 | 0.006 | -0.014** | 0.006 |
| Evaporation | 0.027 | 0.062 | -0.029 | 0.062 |
| $\mathrm{PM}_{2.5}$ | -0.012 | 0.010 | -0.004 | 0.008 |
| $O_{3}$ | -0.004 | 0.006 | -0.004 | 0.005 |
| $SO_{2}$ | 0.033 | 0.040 | 0.030 | 0.038 |
| $\mathrm{NO}_{2}$ | 0.009 | 0.011 |  |  |
| Weekdays | 0.808*** | 0.207 | 1.582*** | 0.211 |
| Holidays | -0.628* | 0.375 | -1.546*** | 0.376 |
| Trend | 0.001*** | 0.000 | 0.000** | 0.000 |
| Adjusted $R^{2}$ | 0.427 |  | 0.565 |  |

Note: *** $p<0.01$, ** $p<0.05$, * $p<0.1$; SE represents the standard error of coefficients.

**Table A4** Factors associated with daily visits by disease (OLS models)

|  | Papular urticaria (N = 1,790) | | Scabies (N = 1,746) | |
| --- | --- | --- | --- | --- |
|  | Coef. | SE | Coef. | SE |
| Visit_1 | 0.184*** | 0.024 | -0.004 | 0.024 |
| Visit_2 | 0.121*** | 0.024 | 0.023 | 0.024 |
| Visit_3 | 0.096*** | 0.024 | 0.007 | 0.024 |
| Visit_4 | 0.073*** | 0.024 | 0.055** | 0.024 |
| Visit_5 | 0.138*** | 0.024 | 0.002 | 0.024 |
| Visit_6 | 0.106*** | 0.024 | 0.096*** | 0.024 |
| Visit_7 | 0.129*** | 0.024 | 0.089*** | 0.024 |
| Visit_8 | -0.008 | 0.024 | 0.005 | 0.024 |
| Visit_9 | 0.004 | 0.023 | 0.010 | 0.024 |
| Temperature_1 | 0.215*** | 0.035 |  |  |
| Temperature_3 |  |  | -0.031* | 0.016 |
| Humidity | 0.003 | 0.021 | 0.013 | 0.011 |
| Wind speed | -0.114 | 0.165 | -0.101 | 0.090 |
| Sunshine | -0.060 | 0.047 | 0.023 | 0.024 |
| Precipitation | -0.024*** | 0.008 | 0.000 | 0.004 |
| Evaporation | -0.052 | 0.081 | 0.083* | 0.042 |
| $\mathrm{PM}_{2.5}$ | -0.005 | 0.013 |  |  |
| $O_{3}$ | -0.007 | 0.007 | -0.002 | 0.004 |
| $SO_{2}$ | 0.023 | 0.053 | 0.072*** | 0.027 |
| $\mathrm{NO}_{2}$ | 0.008 | 0.014 | -0.009 | 0.006 |
| Weekdays | 1.801*** | 0.272 | 0.661*** | 0.143 |
| Holidays | -1.323*** | 0.490 | -0.716*** | 0.262 |
| Trend | 0.000 | 0.000 | 0.001*** | 0.000 |
| Adjusted $R^{2}$ | 0.700 |  | 0.088 |  |

Note: *** $p<0.01$, ** $p<0.05$, * $p<0.1$; SE represents the standard error of coefficients.

## Incorporate CO into the regression models

Consider that CO is a major air contaminant, we incorporate CO into our regression models. The results do not provide strong evidence suggesting that daily visits can be affected by CO; in other words, the coefficient of CO is insignificant (please refer to the red text in Table A5). In contrast, we observe a positive effect of the one-day lagged temperature with a significance level of 1%. This positive effect is consistent with our previous findings in Table 4.

**Table A5** Factors associated with daily visits by adding CO (OLS models)

|  | Total (N = 1,791) | |
| --- | --- | --- |
|  | Coef. | SE |
| Visit_1 | 0.133*** | 0.023 |
| Visit_2 | 0.080*** | 0.024 |
| Visit_3 | 0.075*** | 0.023 |
| Visit_4 | 0.086*** | 0.023 |
| Visit_5 | 0.107*** | 0.024 |
| Visit_6 | 0.116*** | 0.023 |
| Visit_7 | 0.182*** | 0.024 |
| Visit_8 | -0.009 | 0.023 |
| Visit_9 | 0.011 | 0.023 |
| Temperature_1 | 0.279*** | 0.042 |
| Humidity | 0.007 | 0.026 |
| Wind speed | -0.233 | 0.199 |
| Sunshine | -0.066 | 0.056 |
| Precipitation | -0.026*** | 0.009 |
| Evaporation | -0.021 | 0.098 |
| $\mathrm{PM}_{2.5}$ | 0.005 | 0.018 |
| $O_{3}$ | -0.012 | 0.009 |
| $SO_{2}$ | 0.054 | 0.063 |
| $\mathrm{NO}_{2}$ | 0.008 | 0.017 |
| $\mathrm{CO}$ | -1.609 | 1.133 |
| Weekdays | 2.479*** | 0.332 |
| Holidays | -2.045*** | 0.588 |
| Trend | 0.001** | 0.000 |
| Adjusted $R^{2}$ | 0.626 |  |

Note: *** $p<0.01$, ** $p<0.05$, * $p<0.1$; SE represents the standard error of coefficients.

## Seasonal and annual fluctuations in air pollutants

Consider the possible impact of seasonal and annual fluctuations in air pollutants on daily visits. We analyze whether seasonal and annual fluctuations in air pollutants have a significant impact on daily visits, respectively. For brevity, we take the example of analyzing the impact of seasonal fluctuations of $\mathrm{PM}_{2.5}$ on daily visits.

We initially apply a time series decomposition method to break down the original $\mathrm{PM}_{2.5}$ data into trend, seasonality, and residual components. Specifically, we utilize the additive model of the time series decomposition method. The expression of this model can be represented as:

$\mathrm{PM}_{2.5}\left( t \right)=\mathrm{PM}_{2.5}\_T\left( t \right)+\mathrm{PM}_{2.5}\_seasonal\left( t \right)+\mathrm{PM}_{2.5}\_R(t)$,

where $\mathrm{PM}_{2.5}\left( t \right)$ represents the original $\mathrm{PM}_{2.5}$ value at time $t$, $\mathrm{PM}_{2.5}\_T(t)$ denotes the trend component; $\mathrm{PM}_{2.5}\_seasonal(t)$ represents the seasonal component, depicting patterns that repetitively occur over specific periods, i.e., the seasonal fluctuations of $\mathrm{PM}_{2.5}$; $\mathrm{PM}_{2.5}\_R(t)$ stands for the residual component, representing the random fluctuations not explained by the trend and seasonality.

Subsequently, we substitute the original $PM_{2.5}$ value with its seasonal fluctuation term $\mathrm{PM}_{2.5}\_seasonal$ in the regression models. The OLS results indicate that the seasonal components of $PM_{2.5}$ are not statistically significant (please refer to the red text in Table A6).

Similarly, we substitute the original data of $O_{3}$, $\mathrm{SO}_{2}$, and $\mathrm{NO}_{2}$, with their corresponding seasonal components and conduct regression analyses, respectively. The results are presented in Tables A7 – A9. The OLS results indicate that the seasonal components of $O_{3}$, $\mathrm{SO}_{2}$, and $\mathrm{NO}_{2}$ are not statistically significant. Since the analysis process and results for the annual fluctuations of air pollutants are similar to the seasonal fluctuations of air pollutants, we omit them. In summary, the results do not provide strong evidence suggesting that daily visits can be affected by seasonal and annual fluctuations in air pollutants.

**Table A6** Factors associated with daily visits by using the seasonal fluctuations of $\mathrm{PM}_{2.5}$ (OLS models)

|  | Total (N = 1,791) | |
| --- | --- | --- |
|  | Coef. | SE |
| Visit_1 | 0.134*** | 0.023 |
| Visit_2 | 0.082*** | 0.024 |
| Visit_3 | 0.075*** | 0.023 |
| Visit_4 | 0.086*** | 0.023 |
| Visit_5 | 0.107*** | 0.024 |
| Visit_6 | 0.116*** | 0.023 |
| Visit_7 | 0.183*** | 0.024 |
| Visit_8 | -0.008 | 0.023 |
| Visit_9 | 0.012 | 0.023 |
| Temperature_1 | 0.279*** | 0.042 |
| Humidity | -0.000 | 0.025 |
| Wind speed | -0.271 | 0.197 |
| Sunshine | -0.067 | 0.055 |
| Precipitation | -0.024*** | 0.009 |
| Evaporation | -0.001 | 0.097 |
| $\mathrm{PM}_{2.5}\_seasonal$ | 0.043 | 0.048 |
| $O_{3}$ | -0.012 | 0.008 |
| $SO_{2}$ | 0.053 | 0.061 |
| $\mathrm{NO}_{2}$ | 0.000 | 0.013 |
| Weekdays | 2.471*** | 0.332 |
| Holidays | -2.090*** | 0.589 |
| Trend | 0.001** | 0.000 |
| Adjusted $R^{2}$ | 0.626 |  |

Note: *** $p<0.01$, ** $p<0.05$, * $p<0.1$; SE represents the standard error of coefficients.

**Table A7** Factors associated with daily visits by using the seasonal fluctuations of $O_{3}$ (OLS models)

|  | Total (N = 1,791) | |
| --- | --- | --- |
|  | Coef. | SE |
| Visit_1 | 0.134*** | 0.023 |
| Visit_2 | 0.081*** | 0.024 |
| Visit_3 | 0.075*** | 0.023 |
| Visit_4 | 0.086*** | 0.023 |
| Visit_5 | 0.106*** | 0.024 |
| Visit_6 | 0.115*** | 0.023 |
| Visit_7 | 0.182*** | 0.024 |
| Visit_8 | -0.009 | 0.023 |
| Visit_9 | 0.010 | 0.023 |
| Temperature_1 | 0.274*** | 0.041 |
| Humidity | 0.008 | 0.024 |
| Wind speed | -0.204 | 0.192 |
| Sunshine | -0.096* | 0.052 |
| Precipitation | -0.027*** | 0.009 |
| Evaporation | -0.021 | 0.097 |
| $\mathrm{PM}_{2.5}$ | -0.013 | 0.015 |
| $O_{3}\_seasonal$ | 0.008 | 0.025 |
| $SO_{2}$ | 0.059 | 0.063 |
| $\mathrm{NO}_{2}$ | 0.011 | 0.016 |
| Weekdays | 2.458*** | 0.332 |
| Holidays | -2.054*** | 0.588 |
| Trend | 0.001** | 0.000 |
| Adjusted $R^{2}$ | 0.626 |  |

Note: *** $p<0.01$, ** $p<0.05$, * $p<0.1$; SE represents the standard error of coefficients.

**Table A8** Factors associated with daily visits by using the seasonal fluctuations of $\mathrm{SO}_{2}$ (OLS models)

|  | Total (N = 1,791) | |
| --- | --- | --- |
|  | Coef. | SE |
| Visit_1 | 0.134*** | 0.023 |
| Visit_2 | 0.081*** | 0.024 |
| Visit_3 | 0.075*** | 0.023 |
| Visit_4 | 0.086*** | 0.023 |
| Visit_5 | 0.106*** | 0.024 |
| Visit_6 | 0.117*** | 0.023 |
| Visit_7 | 0.183*** | 0.024 |
| Visit_8 | -0.008 | 0.023 |
| Visit_9 | 0.011 | 0.023 |
| Temperature_1 | 0.288*** | 0.042 |
| Humidity | -0.007 | 0.024 |
| Wind speed | -0.281 | 0.197 |
| Sunshine | -0.074 | 0.056 |
| Precipitation | -0.026*** | 0.009 |
| Evaporation | 0.005 | 0.096 |
| $\mathrm{PM}_{2.5}$ | -0.002 | 0.016 |
| $O_{3}$ | -0.010 | 0.009 |
| $SO_{2}\_seasonal$ | 0.034 | 0.245 |
| $\mathrm{NO}_{2}$ | 0.011 | 0.016 |
| Weekdays | 2.454*** | 0.332 |
| Holidays | -2.046*** | 0.588 |
| Trend | 0.001** | 0.000 |
| Adjusted $R^{2}$ | 0.626 |  |

Note: *** $p<0.01$, ** $p<0.05$, * $p<0.1$; SE represents the standard error of coefficients.

**Table A9** Factors associated with daily visits by using the seasonal fluctuations of $\mathrm{NO}_{2}$ (OLS models)

|  | Total (N = 1,791) | |
| --- | --- | --- |
|  | Coef. | SE |
| Visit_1 | 0.135*** | 0.023 |
| Visit_2 | 0.082*** | 0.024 |
| Visit_3 | 0.075*** | 0.023 |
| Visit_4 | 0.086*** | 0.023 |
| Visit_5 | 0.107*** | 0.024 |
| Visit_6 | 0.115*** | 0.023 |
| Visit_7 | 0.182*** | 0.024 |
| Visit_8 | -0.010 | 0.023 |
| Visit_9 | 0.011 | 0.023 |
| Temperature_1 | 0.280*** | 0.040 |
| Humidity | 0.001 | 0.024 |
| Wind speed | -0.287 | 0.191 |
| Sunshine | -0.068 | 0.055 |
| Precipitation | -0.026*** | 0.009 |
| Evaporation | -0.010 | 0.097 |
| $\mathrm{PM}_{2.5}$ | -0.003 | 0.013 |
| $O_{3}$ | -0.011 | 0.009 |
| $SO_{2}$ | 0.071 | 0.059 |
| $\mathrm{NO}_{2}\_seasonal$ | -0.027 | 0.045 |
| Weekdays | 2.465*** | 0.332 |
| Holidays | -2.070*** | 0.586 |
| Trend | 0.001*** | 0.000 |
| Adjusted $R^{2}$ | 0.626 |  |

Note: *** $p<0.01$, ** $p<0.05$, * $p<0.1$; SE represents the standard error of coefficients.
